# Prosthetic Management of an Extensive Maxillary Alveolar Defect with an Implant-Supported Restoration

**Published:** 2013-05

**Authors:** Fariborz Saadat, Ramin Mosharraf

**Affiliations:** 1 Prosthodontist, Dental Implant Research Center, Department of Prosthodontics, School of Dentistry, Isfahan University of Medical Sciences, Isfahan, Iran; 2 Associate Professor, Dental Material Research Center, Department of Prosthodontics, School of Dentistry, Isfahan University of Medical Sciences, Isfahan, Iran

**Keywords:** Dental Implants; Maxilla; Dental Prosthesis; Implant-Supported; Dental Porcelain

## Abstract

Despite the recent developments in peri-implant surgical regenerative procedures, re-establishing the hard and soft tissue contour is still a challenge in cases with severe ridge deficiency. It becomes more difficult when incorrectly placed implants cause screw connections to come out onto the labial surfaces of the teeth. A two-part maxillary implant supported fixed restoration was constructed. The first part was consisted of a screw retained sub-structure that replaced gingival portions of the deficient maxilla and the second part was a cement retained super-structure that reconstructed the anatomical crowns of the lost teeth. In this way awkwardly placed implants did not interfere with the desired esthetic result. Another great advantage was that the alterations or repairs on cemented crowns can easily be carried out without compromising the entire construction.

## Introduction

Extreme ridge resorption in edentulous patients can compromise both esthetics and oral hygiene [1]. Many surgical procedures have been used to re-establish the three dimensional architecture of hard and soft tissue ridge deformities before implant placement [2-4]. Sometimes, patients may be unwilling or unable to tolerate additional surgical procedures to achieve more esthetic results [2]. To reestablish natural crown ratios and natural gingival profiles, alternative treatment approaches must also be considered [5]. Various prosthodontic techniques reported to improve these situations include the using gingiva-colored acrylic resins, a silicone-based tissue-colored material, or removable prostheses [1, 2]. These treatment approaches can reduce the necessity of technique-sensitive surgical procedures that depend on the individual pattern of biologic repair, thereby simplifying and reducing the time and cost of treatment [6].

**Fig1:**
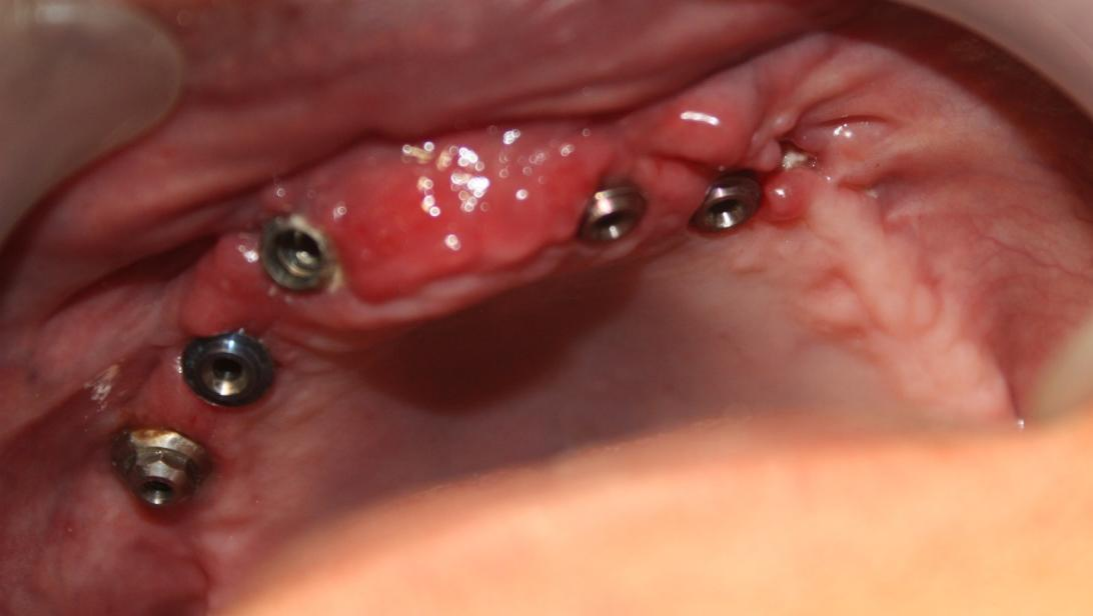
Intra oral view of inserted dental implants

**Fig 2:**
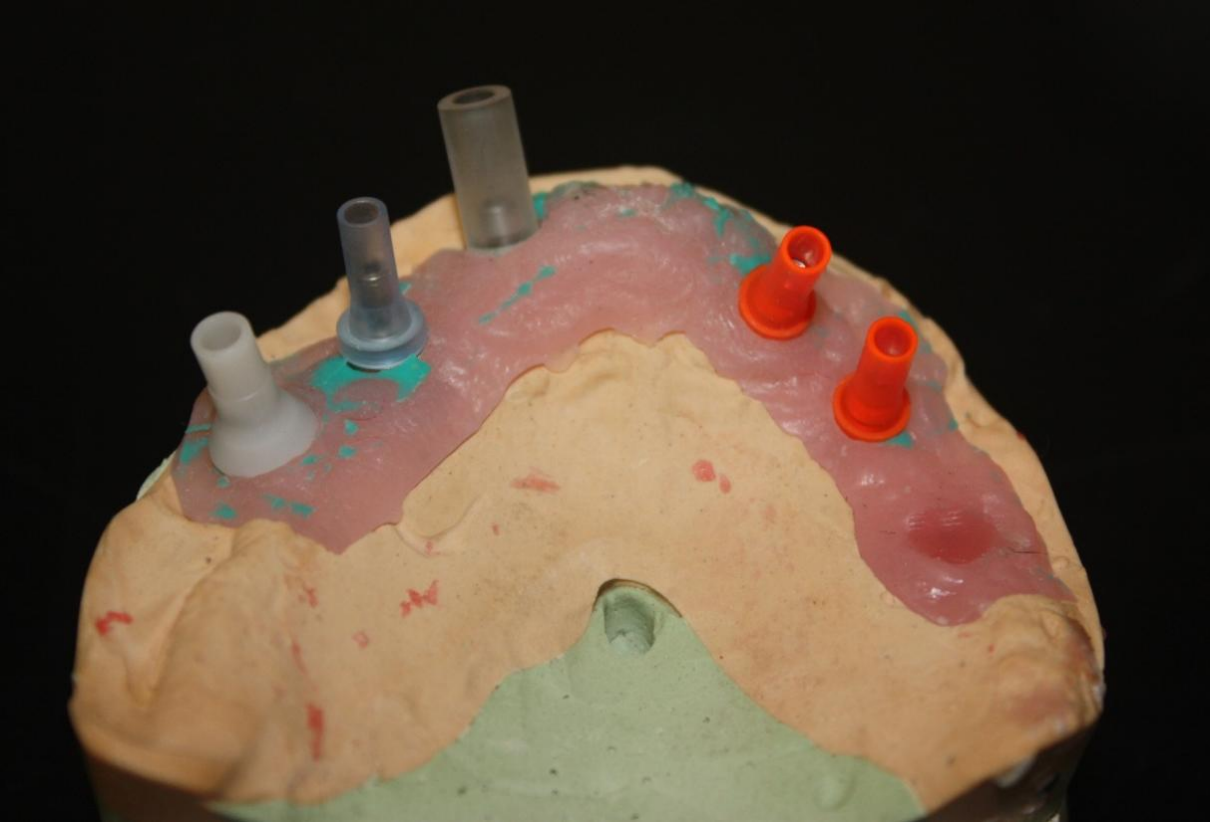
Occlusal view of maxillary cast and plastic copings for making substructure

Furthermore, when implants are incorrectly angled due to ridge resorption, screw connections may come out onto the labial surfaces of the restored teeth [2]. These situations can be corrected by applying gingiva-colored porcelain on the cervical portion of implant-supported restorations [1-3, 6, 7]. However, in these situations creating harmonious mucogingival contours may be simplified by the application of gingiva-colored porcelain onto the cervical collars of metal ceramic restorations [2]. The soft tissue responds well to these ceramic restorations if the patient cleanses as instructed [1]. 

This clinical report describes a two-part implant supported fixed restoration with gingiva-colored ceramic. The first part consisted of a screw retained sub-structure that replaced gingival portions of the deficient maxilla and the second part was a cement retained super-structure that reconstructed the anatomical crowns of the lost teeth.

**TECHNIQUES**

A 55-year-old white man with no significant medical history presented with missing of all maxillary teeth and a provisional maxillary complete denture. In the mandible he had a full arch implant-supported fixed restoration with an acceptable condition. Clinical and radiographic examinations showed the presence of five osseointegrated titanium dental implants (ITI Dental Implant System, Straumann AG, Basel, Switzerland; SwissPlus, Zimmer Dental, CA , USA; Unity, Equinox, Holland) in the maxilla that had to be restored (Fig 1). After primary impression making with an irreversible hydrocolloid (Alginoplast; Heraeus Kulzer, Hanau, Germany) and in the diagnostic jaw relation recording session, it was found that the interocclusal space was more than 15mm. Furthermore, the implant in the area of tooth No. 7 had a buccal angulation (Fig 2). The best choice for this patient was the removable implant supported overdenture. However, the patient refused to wear any type of removable prosthesis. So a two part screw-cemented fixed prosthesis was designed and made. The first part was a metal ceramic screw-type framework with seven custom abutments (according to each dental implant brand) for cementing a fixed bridge on it. The metal frame-works were constructed by a non precious metal (Super 1, Dental Alloy Products, Ca., USA). 

**Fig 3:**
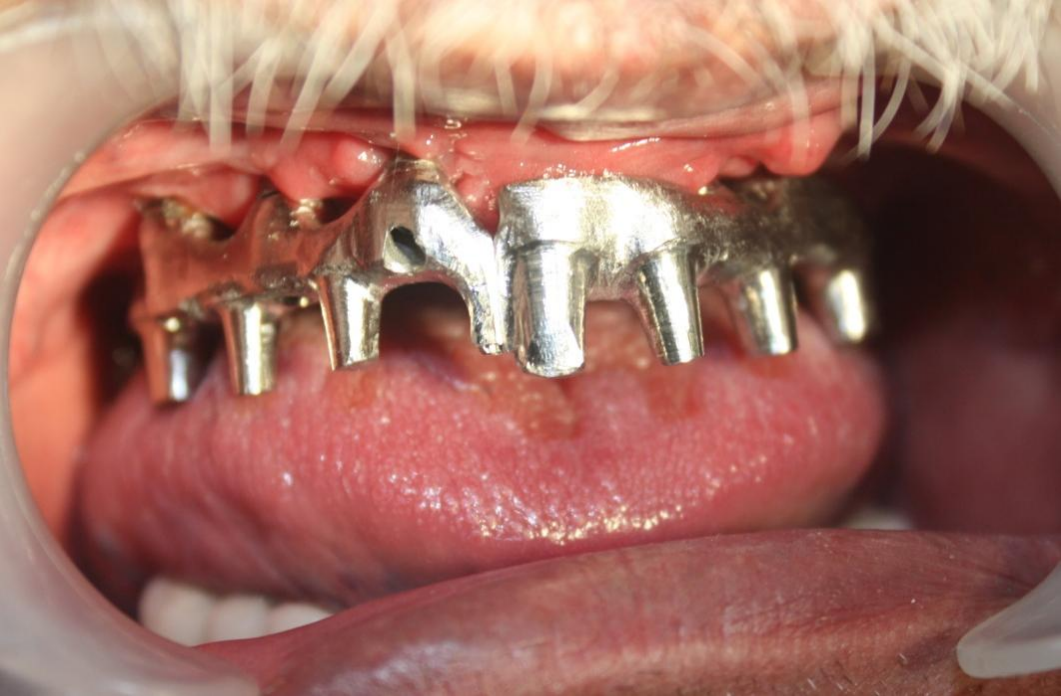
Intra oral view of the first part framework

**Fig 4:**
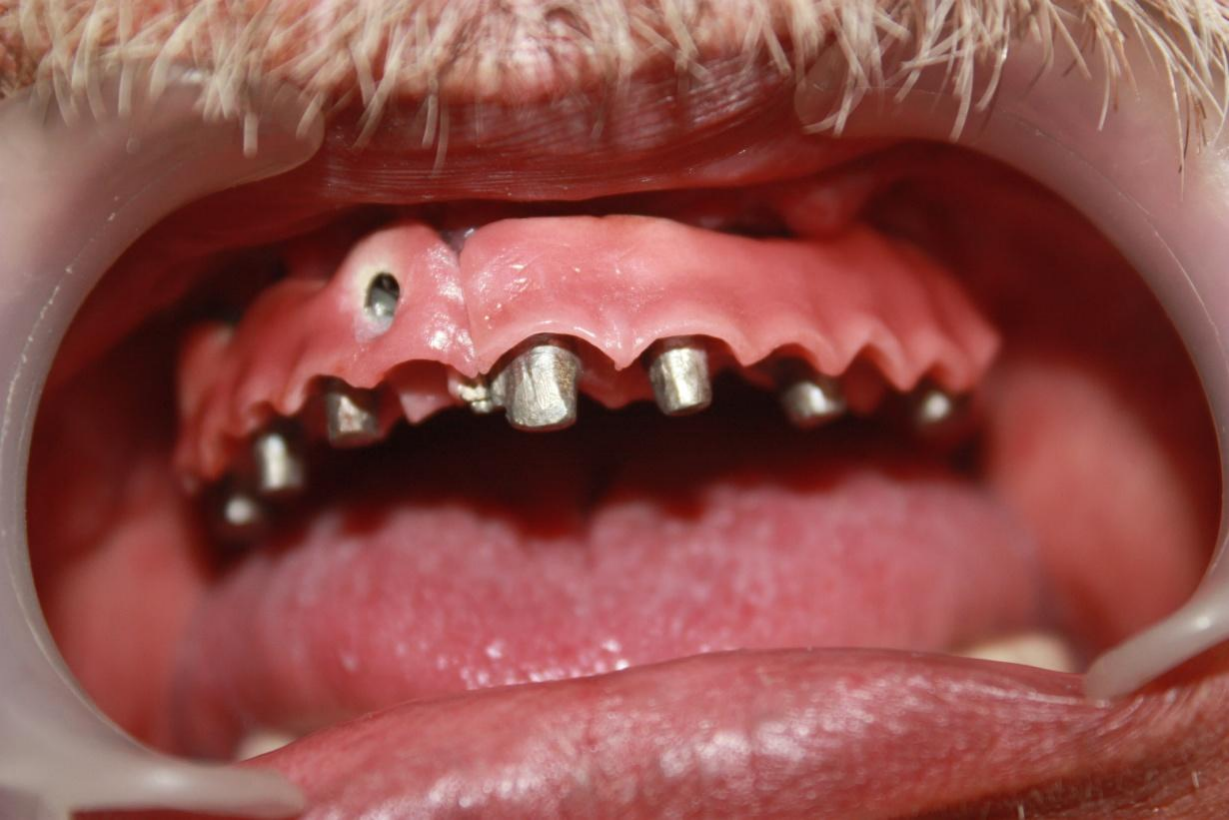
Intra oral view of the completed first part

A semi-precision attachment was incorporated in the midline to overcome the casting problems (Fig 3) therefore, awkwardly placed implants especially in the area of tooth No.7 caused no interference with the desired esthetic result. The porcelain gingival surfaces (EX-3, Noritake dental Co., Aichi, Japan) were convexly designed to ensure the best possible cleaning (Fig 4). 

**Fig 5:**
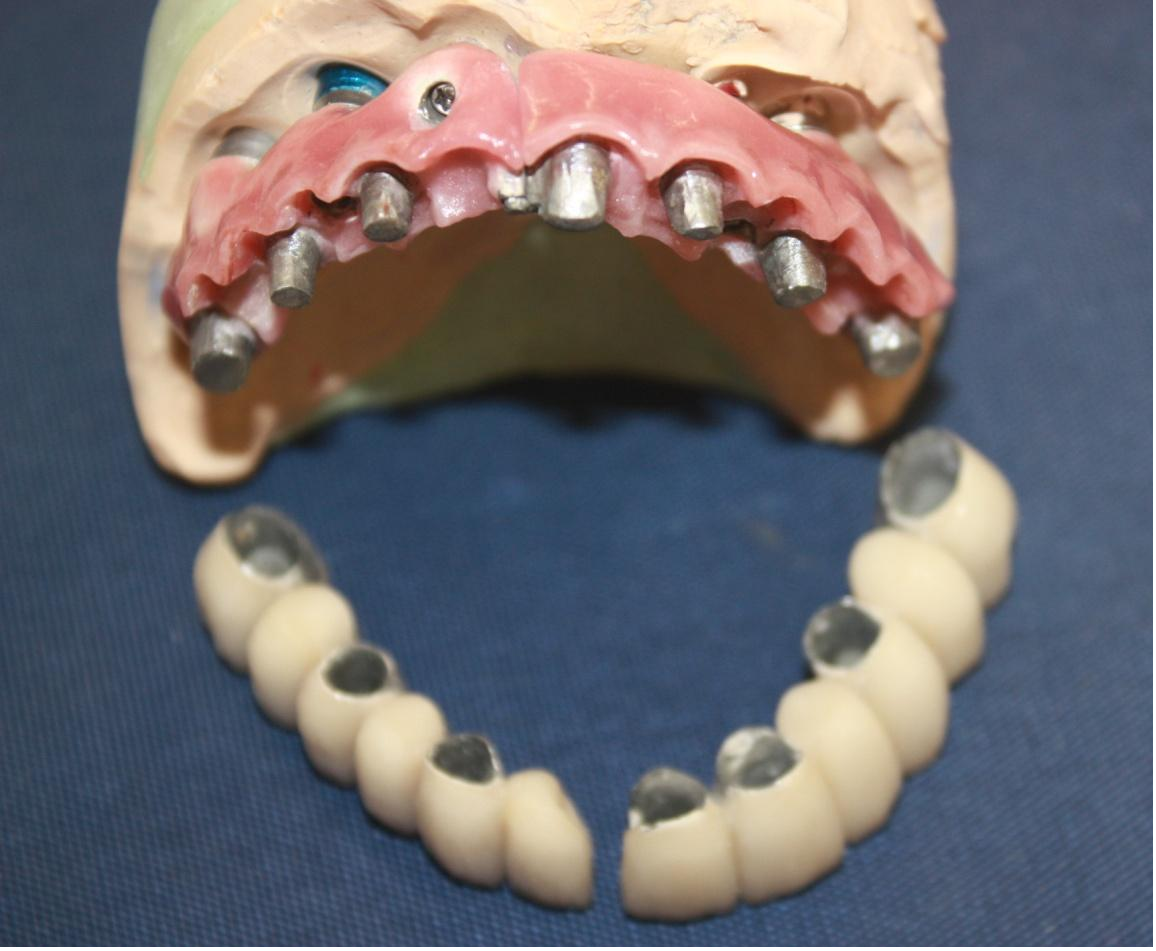
The final work before inserting in the mouth

**Fig 6:**
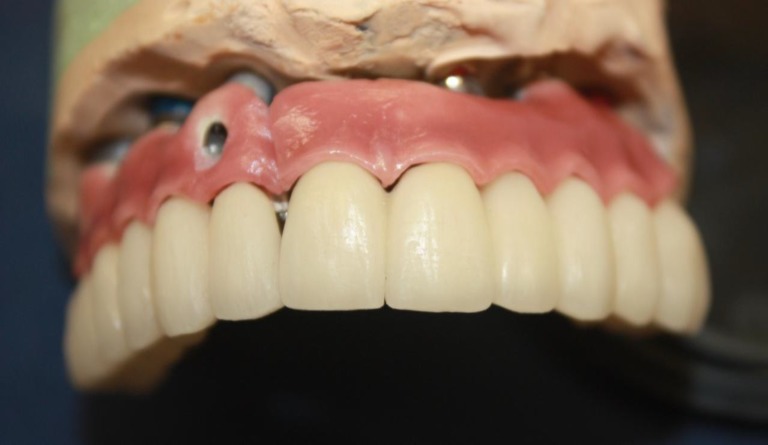
The assembled final work before inserting in the mouth

The secondary part was made as a two section cemented conventional metal ceramic fixed partial denture (Fig 5 and 6).

The impression was taken on fixture level with transfer type impression coping with A-silicon impression material (Panasil, Kettenbach GmbH & Co. KG, Eschenburg, Germany). Master casts (Vel-Mix, Sybron, Kerr, USA) were fabricated. For designing this type of prosthesis, a diagnostic teeth arrangement was first set on the record base for evaluation of occlusal vertical dimension, interarch distance, centric relation, and the assessment of the patient’s esthetic desires. This fully contoured wax-up was evaluated intraorally and modified until a natural and esthetic appearance was established. A silicon matrix was used to transfer the contour of the tried wax-up to the definitive prosthesis. Because forming the gingival porcelain without having final crown contours was very difficult, the porcelain crowns of the secondary part were made at first (according to silicon matrix) and then layering of the gingival porcelain was performed, whereas the secondarpart had been inserted on the custom abutments.

**Fig 7:**
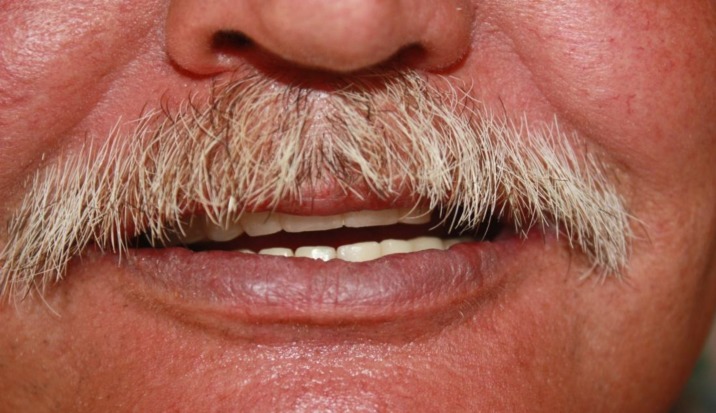
Intra oral view of cemented fixed restorations

The occlusion of the maxillary prosthesis was adjusted to achieve simultaneous centric relation contact and canine guidance occlusion [8, 9]. In the delivery session, the gingival parts were screwed on the implants (with 30 Newton Torque) and periapical radiographs were taken for the examination. Then, the screw opening in the buccal area of tooth No. 7 was filled with pink dental composite restorative material (PermaFlo^®^ Pink, Ultradent Products Inc., USA). In the next step, the second part was provisionally cemented (Temp Bond- Kerr Italy-Salerno- Italy). At the end, the patient was particularly impressed with the perfect fit and natural appearance of this bridge (Fig 7). The follow-up sessions were performed and patient maintenance and oral hygiene were evaluated regularly.

## Discussion

Understanding the patient’s clinical requirement is essential before soft-tissue replacement with either fixed or removable prostheses [10]. Fixed implant supported restorations have many advantages in comparison to removable prostheses [11, 12]. Fixed prostheses have some benefits such as excellent comfort, more self-confidence for the patient and less maintenance visits. 

However, replacing a large tissue defect and creating ideal contour is easier with removable prostheses [13]. Generally, fixed implant prostheses are contraindicated for maxillary edentulous patients because of deficient lip support, limited access for hygiene, phonetic difficulties due to the escape of air, and esthetic complications due to misalignment between the long axes of the replacement teeth and inserted implants [14]. However, for the patients who desire fixed restorations using gingiva colored porcelain can be useful. With these prostheses lip support and access for maintaining oral hygiene can be easily obtained [13]. Some authors [11, 15, 16] reported phonetic complications in conventional fixed implant maxillary prostheses due to the escape of air. In these situations, adding gingival porcelain can fill the spaces and return the standard speech patterns [13]. In this case, the opposing occlusion was a fixed implant supported full arch restoration and its occluding surface was made from porcelain. So we did not make a resin metal hybrid restoration with acrylic occlusal surfaces due to probable wear results [17]. Making conventional metal-ceramic restorations in situation that implants do not align within the long axis of the replacement teeth, is very difficult [13]. 

By using this two-part fixed restoration, the prosthetic teeth were positioned in such a way that did not need to be positioned directly over the implants. The other benefit of using a fixed prosthesis is the higher masticatory ability for the patients [11]. Another great advantage is that the alterations or repairs on cemented crowns can easily be carried out without compromising the entire construction. A similar prosthetic design named “Prettau Zirconia” was introduced to the market by a commercial factory [18]. But in some situation that there is no CAD/CAM equipped laboratory available; using this handmade metal ceramic restoration can be useful. So, one advantage of this treatment approach is making fixed prosthesis for patients who do not like removable dentures and making conventional fixed implant supported prosthesis is not possible. Another advantage is managing buccally angulated implants. In comparison to similar CAD/CAM designed zirconia bridges, the cost of this treatment approach is very low and making this type of prosthesis is possible with conventional equipment that are available in any dental laboratory. Disadvantages of this two-part fixed implant supported restoration as compared to removable over-denture is the increased cost because of needing more implants and more laboratory expenses due to required gingival screw-type substructure. Although initial expenses are greater, the lower maintenance required in these restorations could result in reduced long-term expense [13, 14].

## CONCLUSION

Re-establishing the hard and soft tissue contour is a challenge in patients with extensive ridge resorption. It becomes more difficult when incorrectly placed implants cause screw connections to come out onto the labial surfaces of the teeth. Here, we constructed a two part maxillary implant supported fixed restoration. The first part consisted of a screw retained sub-structure that replaced gingival portions and the second part was a cement retained super-structure that reconstructed the anatomical crowns of lost teeth. This restoration provides some advantages over removable overdentures including excellent comfort, more self-confidence for the patient and less maintenance visits.

## References

[1] Kim TH, Cascione D, Knezevic A, Nowzari H (2010). Restoration using gingiva-colored ceramic and a ridge lap pontic with circumferential pressure: a clinical report. J Prosthet Dent..

[2] Kamalakidis S, Paniz G, Kang KH, Hirayama H (2007). Nonsurgical management of soft tissue deficiencies for anterior single implant-supported restorations: a clinical report. J Prosthet Dent..

[3] Horvath SD, Kohal RJ (2011). Rehabilitation of an extensive anterior explantation defect--a case report. Quintessence Int..

[4] Etoz OA, Demetoglu U, Ocak H (2012). New Method to Increase Inter-Alveolar Height with Preservation of Crestal Cortical Bone for Implant Treatment. J Oral Implantol.

[5] Khatami AH, Al-Ajmi M, Kleinman A (2006). Preservation of the gingival architecture with the scalloped implant design: a clinical report. J Oral Implantol..

[6] Garcia LT, Verrett RG (2004). Metal-ceramic restorations--custom characterization with pink porcelain. Compend Contin Educ Dent..

[7] Sleiter R, Klimek K, Jenni S (2011). [Implant supported anterior crowns customized by computer-aided design. State of the technique and case report]. Schweiz Monatsschr Zahnmed.

[8] Klineberg IJ, Trulsson M, Murray GM (2012). Occlusion on implants - is there a problem?. J Oral Rehabil.

[9] Koyano K, Tsukiyama Y, Kuwatsuru R (2012). Rehabilitation of occlusion - science or art?. J Oral Rehabil.

[10] Buser D, Martin W, Belser UC (2004). Optimizing esthetics for implant restorations in the anterior maxilla: anatomic and surgical considerations. Int J Oral Maxillofac Implants.

[11] Goodacre CJ, Kan JY, Rungcharassaeng K (1999). Clinical complications of osseointegrated implants. J Prosthet Dent..

[12] Sones AD (1989). Complications with osseointegrated implants. J Prosthet Dent..

[13] Priest GF, Lindke L (1998). Gingival-colored porcelain for implant-supported prostheses in the aesthetic zone. Pract Periodontics Aesthet Dent..

[14] Heydecke G, Boudrias P, Awad MA, De Albuquerque RF, Lund JP, Feine JS (2003). Within-subject comparisons of maxillary fixed and removable implant prostheses: Patient satisfaction and choice of prosthesis. Clin Oral Implants Res..

[15] Eckert SE, Carr AB Implant-retained maxillary overdentures. Dent Clin North Am.2004 Jul.

[16] Noriko T, Kento T, Tsuneji O, Motohiro M, Kozue M, Makoto S (2003). [A clinical study on unfavorable cases of dental implant]. Kokubyo Gakkai Zasshi..

[17] Hahnel S, Behr M, Handel G, Rosentritt M (2009). Two-body wear of artificial acrylic and composite resin teeth in relation to antagonist material. J Prosthet Dent..

[18] zirkonzahn (2012). Why using Prettau Zirconia?. http://www.zirkonzahn.com/en/prettau-zirconia.aspx.

